# Supplementary material for: Spectrum of Atazanavir-Selected Protease Inhibitor-Resistance Mutations
Source: Pathogens. 2022 May 5;11(5):546. doi: 10.3390/pathogens11050546 (PMC9148044; doi:10.3390/pathogens11050546)
Supplement: Supplementary file 1 [file pathogens-11-00546-s001.zip › Table S2.pdf]

Table S2. Nonpolymorphic PI treatment-selected mutations (NP-TSMs) occurring in >=1 sequences from patients receiving boosted or unboosted atazanavir (ATV) as their first PI.

NP-TSM[1]	% in Patients with NP-TSMs (n=149)	% With DRMs[2]	% In Patients receiving boosted or unboosted ATV (n=1497)	% In PI-naïve Patients (n=61597) [1]
89T	34.9	71.2	3.5	0.00
55R	15.4	87	1.5	0.26
85V	11.4	88.2	1.1	0.00
71I	9.4	100	0.9	0.10
34Q	7.4	81.8	0.7	0.08
22V	4.7	57.1	0.5	0.07
92R	4	50	0.4	0.01
95F	3.4	100	0.3	0.16
34D	3.4	80	0.3	0.02
66V	3.4	80	0.3	0.09
72L	3.4	60	0.3	0.00
34N	2.7	100	0.3	0.06
53I	2.7	75	0.3	0.09
66F	2.7	100	0.3	0.33
72K	2.7	75	0.3	0.02
45I	2	100	0.2	0.00
79A	2	66.7	0.2	0.06
53Y	1.3	100	0.1	0.00
71L	1.3	100	0.1	0.01
51A	1.3	100	0.1	0.12
10Y	1.3	100	0.1	0.00
91S	1.3	100	0.1	0.06
43Q	1.3	0	0.1	0.02
55N	1.3	100	0.1	0.00
43N	1.3	0	0.1	0.01
96S	1.3	0	0.1	0.01
89P	0.7	100	0.1	0.00
67L	0.7	100	0.1	0.02
10R	0.7	100	0.1	0.05
33M	0.7	100	0.1	0.02
43I	0.7	100	0.1	0.00
45Q	0.7	100	0.1	0.01
74E	0.7	100	0.1	0.00

[1]NP-TSMs were defined in the previous study, Rhee et. al, 2016 and the prevalence of each NP-TSM in PI-naïve patients was reported in the same publication.

[2]DRMs (drug resistance mutations) were defined as those with a Stanford HIV drug resistance program penalty score for ≥1 PI.
