# Supplementary material for: Spectrum of Atazanavir-Selected Protease Inhibitor-Resistance Mutations
Source: Pathogens. 2022 May 5;11(5):546. doi: 10.3390/pathogens11050546 (PMC9148044; doi:10.3390/pathogens11050546)
Supplement: Supplementary file 1 [file pathogens-11-00546-s001.zip › Table S3.pdf]

Table S3. Studies in PubMed containing sequences from previously PI-naïve patients receiving boosted or unboosted atazanavir (ATV) for which the sequences were not available.

AuthorYr	Trial	# Total ATV	# Boosted ATV	# Unboosted ATV	% Reported DRMs[1]	Reported DRMs (n)
Dolling13		322	251	71	1.9	I50L (3); I84V (2); N88S (1)
Wallis20	ACTG A5288	170	170	0	40.0	Not Listed
Lambert-Niclot18		113	95	18	3.5	L33F+I50L (1); M46I+N88S (1)<br>G48V+I54L+G73T+V82T+I84V (1);<br>L33F+F53L+I54L+A71V+V82T+I84V+L90M (1);
Marcelin14		90	90	0	3.3	N88S (2); I50L (1)
Teófilo16	REMAIN	85	85	0	0	
Molina10	CASTLE	39	39	0	7.7	I50L (1); V32I+M46I+I84V (1)<br>L10F+V32I+K43T+M46I+A71I+G73S+I85I/V+L90M (1);
Chawana17	ATF	28	28	0	35.7	I50L (1); A71T (1); L90M (1); M46I (1);<br>Q58E+V82M (1); A71I/T+N88S+L10V (1);<br>L10F+M46I+Q58E+A71I+I84V (1);<br>I50L+V82M+V32I+L24I+N83D (1);<br>M46I+I50L+L10V+L33F+I47V+A71V+G73C/S+V82A (1);<br>V82M+A71V+L24I+K43T+F53L+I54V+T74P (1)
Soriano11	ARTEN	28	28	0	0	
Palumbo18	HPTN 052	28	2	26	0	
Gallant13	GS-US-216-0114	24	24	0	0	
Kulkarni17	WAVES	21	21	0	0	
Kulkarni14	GS-US-236-0103	19	19	0	0	
Kumar09	ACTION	16	16	0	0	
Stebbing07		16	16	0	0	
Malan10	BMS AI424-089	10	2	8	40.0	N83N/D (1); I50IL (1); I50I/L+G73G/S (1);<br>I50L+N88N/S+K20K/I/T+L33F (1)
Slama16	IMEA 040 DATA	9	9	0	22.2	M46I+N88S (1); D30N+N88D (1)
Elion08		8	8	0	0	
Squires10	ARIES	8	8	0	0	
Castain19		6	6	0	0	

Kozal12	SPARTAN	6	1	5	0	
Martinez15	ATADAR	6	6	0	0	
Mills13	A4001078	6	6	0	0	
Obasa20		6	6	0	33.3	Not listed
Elion08		5	5	0	0	
Landman10		4	0	4	0	
Orrell17	ARIA	4	4	0	0	
Pujari18		4	4	0	25.0	M46I+N88T (1)
Karlström07		3	3	0	0	
Ross10	ALERT	3	3	0	0	
Lambert-Niclot10		2	2	0	0	
Bartlett13		1	1	0	0	
Mahomed20		1	1	0	0	
[1]DRMs (drug resistance mutations) were defined as those with a Stanford HIV drug resistance program penalty score for ≥1 PI.						
