# Supplementary material for: Spectrum of Atazanavir-Selected Protease Inhibitor-Resistance Mutations
Source: Pathogens. 2022 May 5;11(5):546. doi: 10.3390/pathogens11050546 (PMC9148044; doi:10.3390/pathogens11050546)
Supplement: Supplementary file 1 [file pathogens-11-00546-s001.zip › Text S1.pdf]

Text S1. The Euresist Network Study Group is formed by:

Francesca Incardona,
Rolf Kaiser,
Thomas Lengauer,
Anders Sönnerborg,
Maurizio Zazzi,
Ana Abecasis.
Marina Bobkova,
Carole Devaux,
MIlos Parczewski,
Nico Pfeifer,
Michal Rosen-Zvi,
Murat Sayan,
Antonia Bezenchek,
Giulia DI Teodoro
